# Predictors for CD4 cell count and hemoglobin level with survival time to default for HIV positive adults under ART treatment at University of Gondar Comprehensive and Specialized Hospital, Ethiopia

**DOI:** 10.1186/s13104-023-06625-3

**Published:** 2023-12-02

**Authors:** Nurye Seid Muhie, Awoke Seyoum Tegegne

**Affiliations:** 1 Mekdela Amba University, Tulu Awuliya, Ethiopia; 2 Bahir Dar University, Bahir Dar, Ethiopia

**Keywords:** CD4 cell count, Hemoglobin level, Time to default, Bayesian joint model, HIV/AIDS

## Abstract

**Background:**

HIV/AIDS is the most known powerful risk factor for morbidity and mortality in the world. The greatest biological markers in HIV patients are CD4 cell count and hemoglobin level, as they are independent predictors of survival of HIV patients. The objective of this study was to investigate the common socio-demographic, clinical, and behavioral Predictor’s affecting the CD4 cell count, and hemoglobin level with survival time to default from ART treatment among HIV positive adults under ART treatment at university of Gondar comprehensive and specialized hospital, North-west Ethiopia.

**Method:**

This study was conducted at University of Gondar comprehensive specialized hospital by using a retrospective cohort follow up study design. The source of data in this study was secondary data obtained from patients chart. Bayesian joint models were employed to get wide-ranging information about HIV/AIDS progression.

**Result:**

From a total of 403 HIV positive adults, about 44.2% were defaulted from therapy and the rest were actively followed ART treatment. The estimate of the association parameter for the current true value of CD4 cell count (${\alpha }_{1}$), and hemoglobin level (${\alpha }_{2}$), trend of CD4 cell count (${\alpha }_{2}$) and hemoglobin level (${b}_{2}$) is positive. Positive values indicating that the higher CD4 cell count and hemoglobin level is related with the higher time of defaulting from ART. Predictor’s hematocrit, weight, platelet cell count, lymphocyte count, sex, adherence, and WHO clinical stage were joint determinate risk factors affecting CD4 cell count, hemoglobin level and time to default at 5% level of significance.

**Conclusion:**

Current study results revealed that hematocrit, weight, BMI, platelet cell count, lymphocyte count, sex (female), and good treatment adherence were significantly associated with higher CD4 cell count, hemoglobin level and time to default while having advanced WHO clinical stage-IV had significantly decreased CD4 cell, hemoglobin level, and time to default from treatment. Patients with HIV should be given special attention based on these important factors to improve their health and prolong their lives.

**Supplementary Information:**

The online version contains supplementary material available at 10.1186/s13104-023-06625-3.

## Introduction

HIV/AIDS is the most known powerful risk factor for morbidity and mortality in the world [1]. In 2020 37.6 million people were living with HIV, 1.5 million were newly infected, 690 000 people died from AIDS related illnesses, and 27.4 million people were accessing antiretroviral therapy (ART) increased from 7.8 million in 2010 [2].

Eastern and Southern Africa region has been strongly hit by HIV/AIDS [3]. UNAIDS report indicated that in the year 2020 an estimated 20.6 million people living with HIV (PLWHIV) were found in Eastern and Southern Africa region and also approximately 670,000 new HIV infection patients, 600,000 age greater than 14 years and 310,000 AIDS related deaths [2].

Ethiopia has also one of the largest HIV infected population among sub-Saharan Africa countries [4]. The disease gradual decline with an estimated 669,236 for the year 2019 to 622,326 in 2020,also adult HIV new infection declined from 11,613 to 8,921 for the year 2019 and 2020 respectively [5]. Even though, HIV prevalence in Ethiopia decreased from 3.3% in 2000 to 0.9% in 2017 and AIDS related deaths from 83,000 deaths in 2000 to 15,600 in 2017 [6], the virus has still a significant epidemic burden [7].

Excessive progression of HIV leads to decreased of immune response [8] as well as blood hemoglobin levels [9] and substantially associated with decreased survival of patients. This indicated CD4 cell count and hemoglobin level are the greatest biological markers in HIV patients and independent indicators of survival time to default from ART treatment.

Defaulting is Patients who did not returned to care ART treatment after their last longitudinal visit time [10]. There are a variety of reasons why people may be defaulted from ART treatment: it could be due to death, transferred out, lost to follow-up, poor health circumstances such as low CD4 cell counts [10, 11] and hemoglobin levels. People living with HIV (PLWHIV) may be also defaulted due to psychological, family, and social problems. The most common issues associated with those problems are; lack of motivation and confidence, depression in patients and families, family conflicts, rejection of patients by family members, social isolation, lack of active and impressive support communities [12].

CD4 cells are a major target for HIV patients, the virus binds to the surface of CD4 cells, enters them, and either replicates immediately, killing the cells in the process, or remains in a resting state, replicating later [13]. For healthy adult peoples CD4 cell count in the normal range between 500 and 1500 cells/mm^3^.Otherwise, an indication of immunological failure of patients whose CD4 cell count less than 500 cells/mm^3^and HIV strongly affects the immune system below 100 cells/mm^3^ [14].

The significance of hemoglobin level is a standalone predictive indicator of HIV progression and death beginning with ART [9]. Hemoglobin concentration for men and women in normal health status varies in sex, ethnicity, physiological status [15], lifestyle, and socioeconomic status. Contrariwise, Hemoglobin concentration less than 13 g/dL and 12 g/dL for men and women respectively can be a sign of an infection disease like anemia [16], cancer, chronic obstructive pulmonary disease (COPD), congenital heart disease, emphysema, polycythemia Vera, and pulmonary fibrosis. Associated with those infectious diseases, patient’s leads to reduction of immune system and defaults from ART treatment through variety of reasons.

On the other hand adult HIV positive patients can be low concentration of CD4 cell count and hemoglobin level below the expected healthy adults strongly leads to high HIV progression and patient’s defaults from ART treatment. CD4 cell count, hemoglobin level, and survival rates can be drastically improved by early ART initiation [17]. However, many patients are defaulting from ART due to substantial variation in CD4 cell count and hemoglobin level [18] and a dramatic influence on mortality and morbidity [19].

Most of the previous literature conducted with CD4 cell count, hemoglobin level and time to default separately. Some of the studies done with separately indicated that predictors associated with default from ART were low body mass index, WHO clinical stage-IV, opportunistic infections, weight, alcohol drinker, rural residence [11], lack of transport, unemployment occupation [20], male sex, age (15–24 and ≥ 45 years) [21], bedridden functional status, having an unknown HIV disclosure status, dependent patients for source of food, patients whose partners were HIV negative, and patients that fear stigma [22]. Likewise, other study showed that hemoglobin level of the patients was statistically significant predictors for default from ART. Contrariwise other hematological parameters such as white blood cell (WBC), lymphocyte, monocyte, eosinophil, neutrophil, and basophil counts were no statistically significant changes for defaulters of ART [18].

Independent predictors for CD4 cell count were age, weight, baseline CD4 cell count, cell phone ownership, visit time, marital status, residence, and level of disclosure of the disease to family members [23], household income, WHO clinical stage, ART adherence [24],opportunistic infections [25].

Further study also revealed that HIV Patients were older age, female sex, hepatitis C virus coinfection, higher virial load, 1c ART treatment, illicit drug and BMI < 18.5 kg/m^2^ were associated with low hemoglobin level; on the contrary BMI between 25 to < 30 kg/m^2^ or BMI ≥ 30 kg/m^2^, smoker and alcohol addicted were associated with higher hemoglobin level overtime [26]. Other study indicated that, weight, CD4 cells < 350/mm^3^ [27], tuberculosis co-infection, advanced WHO stage [28], other opportunistic infections, having poor adherence to ART, rural residence, and eating non-diversified foods were significantly associated with low hemoglobin level [29].

Numerous studies were conducted as some mentioned above for analyzing predictors of CD4 cell count, hemoglobin level and time to default separately that do not considered dependencies between these two different data types (longitudinal and time-to-event data) jointly. However, Bayesian joint model simultaneously estimates both the linear mixed-effects model for longitudinal data and Cox PH models for survival data and it is better suited for analyzing such data because they estimate jointly the time-to-event outcome conditional upon the longitudinal outcome [30].

Therefore, as far as the authors’ knowledge is concerned, there is sparse of a study now conducted for CD4 cell count, hemoglobin level and survival time to default from ART treatment jointly among adult HIV positive patients. This study was undertaken with the objective was to identify common socio-demographic, clinical, and behavioral predictors affecting the CD4 cell count, and hemoglobin level with survival time to default from ART treatment among HIV positive adults under ART treatment at university of Gondar comprehensive and specialized hospital (UGCSH), Gondar Ethiopia.

## Materials and methods

### Study area

This study was conducted at University of Gondar comprehensive specialized hospital (UGCSH), Gondar, Ethiopia which was found in Gondar city. Since, these study area selected with regarding to large no of adult HIV positive patients to obtain relevant information about CD4 cell count, hemoglobin level, survival event, and related covariate.

### Study population

The study population for this study was all adult HIV positive patients treated at UGCSH.

### Source of data

Secondary data source obtained by reviewing of the patient’s chart under the follow-up period.

### Study period

Patients who follow-up treatment from September 2015–March 2022 G.C.

### Inclusion criteria

This study was considered adult HIV positive patients who attended at least a minimum of two visits time for longitudinal response, patients whose age ≥ 15 years, and started treatment within follow-up study period.

### Exclusion criteria

This study doesn’t was considered adult HIV positive patients who attended only one visits time for longitudinal response, patients whose age < 15 years, and started treatment without follow-up study period.

### Data collection procedure

The data collection procedure in this retrospective cohort study was based on medical registration number (MRN) and patient’s chart. First selected patients chart by using their MRN from electronic database system and from the review of patients chart based on inclusion criteria the necessary information were retrieved by two ART data clerks who were trained on ART data management.

### Data collection quality

In order to maintain the quality of collected data, one day intensive training was given for data collectors based on the aim of this study. At that time, adequacy of the checklist was evaluated and ambiguous questions are modified before the actual data collected. The necessary amendments are made on the final data collection format for completeness and consistency and the full formats are checked by ART data management.

### Variables in the study

#### Response variable

The response variable for this study were CD4 cell count/mm^3^, hemoglobin (Hgb) level/dL and time to default from ART treatment. The repeated biomarker of adult HIV positive patients CD4 cell count and hemoglobin level was measured within 6 months interval. In data collection the visit time for CD4 cell count and hemoglobin level was not the same for each patient. Therefore, we approximate every six month and the necessary information was recorded starting from baseline month, 6 month, 12 month, 18 month, 24 month, 30 month,36 month, 42 month, 48 month, 54 month, 60 month, 66 month and 72 month.

Time to default of survival outcome was measured in month by subtracted the month of started ART treatment from month of defaulted occurrences.

#### Independent variable

The Independent variables in the current study were gender, age in year, level of education, disclosure status, tobacco addiction, alcohol addiction, weight in kg, body mass index in kg/m^2^, functional status, treatment adherence, WHO clinical stage, TB screen, opportunistic infection (OIs) other than TB, other comorbid condition (OCC), hematocrit, White Blood Cell, Red Blood Cell, platelet cell count, lymphocyte count, and monocyte count.

### Operational definition

Gender: can be classified in to male and female categories.

Age in year: Age in year can be classified as 15–24, 25–34, 35–44, > 44 years.

Level of education: for adult patients educational level can be categorized as Non-educated, Primary, Secondary, and Tertiary education.

Disclosure: is sharing your HIV status for families, friends, or a health care setting can help with the stresses of living with HIV. However, deciding whom to tell and how to tell them can be complicated and difficult because the important thing is that you choose a place that is comfortable for HIV patients.

Tobacco addiction and alcohol addiction: both are considered as substance use disorders, which are problematic patterns closely associated with HIV and other sexually transmitted diseases.

Weight: HIV-infected individuals on ART have gained weight, which has been attributed to a return to health condition where appetite increases and more food is ingested along with minimal physical activity.

Body mass index: body mass index (BMI) may contribute somewhat on drug metabolism and thus affects antiretroviral therapy (ART) effectiveness.in any cross-sectional or longitudinal study, baseline BMI was calculated as body weight in kilograms/(height in meters)**.**

Functional status: According to WHO standards, the functional status was rated as follows: (I) Working: Capable of carrying out routine tasks inside or outside the home. (II) Ambulatory: Capable of performing Activities of Daily Living but unable to perform any employment. C) Bedridden: Unable to carry out Activities of Daily Living.

TB screen: HIV-positive individuals may or may not be TB in some conditions. Nevertheless, HIV patients have an increased risk of developing tuberculosis (TB) than non-HIV individuals. This is because HIV impairs immunity, making it more difficult for the body to fight against TB germs.

WHO Clinical stage of patients: can be classified as: Stage I (asymptomatic disease), Stage II (mild disease), Stage III (advanced disease) and Stage IV (severe disease).

OIs (Opportunistic infectious) disease: patients affected by different AIDS related infections like bacterial pneumonia, salmonella septicemia, fever, diarrhea, candidiasis, toxoplasmosis, wasting syndrome, and other.

OCC (other comorbid condition): HIV Patient’s would be affected by different chronic illness like diabetes, hypertension, cancer disease, and anemia.

Treatment adherence of patients: was classified based on WHO classification: poor adherence was classified as the percentage of the missed dose was < 85%, fair adherence the percentage of the missed dose was 85%–94%, and good Adherence was classified as the percentage of missed dose ≥ 95% [31].

Hematocrit, White Blood Cell, Red Blood Cell, platelet cell count, lymphocyte count, and monocyte count: both can be considered as hematological parameters among HIV positive patients.

### Method of data analysis and models used in the current investigation

In this study, Secondary data was collected by Microsoft excel, the collected data imported in to SPSS version 26 for data management and finally imported in to R- software Version 4.1.3 for statistical data analysis by considered 5% level of significance.

### Statistical models

In this study, the authors used Bayesian bivariate Linear mixed effect model (LMEM) to investigate the determinant factors that can affect CD4 cell count and hemoglobin level. Cox PH model had been used to determinant factors that affect the survival time to default from ART treatment. Finally, statistical joint model analysis was used to assess the impact of repeated measure CD4 cell count and hemoglobin level with time to default from ART treatment. Based on the complexity of the data and the desired objectives of the study, the authors considered the following three different statistical models:

- Bayesian bivariate LMEM was used for continuous response variable for the repeated measure hemoglobin level and CD4 cell count.
- Cox PH model was used for survival time to default from ART treatment.
- Joint model was used for repeated measure hemoglobin level and CD4 cell count with survival outcome.

## Results

### Socio-demographic characteristics of HIV patients

Females made up more than half of the patients in this study (64.5%), of which 55.4% defaulted from ART treatment during the follow-up period. Regarding age in years of patients, a large percentage of patients 182 (45.2%) were aged between 25 and 34 years, of which 135 (74.2%) defaulted throughout the study period. Furthermore, in terms of functional status, 31.64%, 77.2%, 48.2% were working, ambulatory, and bedridden patients respectively defaulted from ART treatment. Similarly, among 220 (54.6%) urban participants, 65 (29.5%) defaulted from ART treatment. Regarding the level of education, 139 (34.5%) had attended primary school education and out of them more than a half (54.64%) defaulted from ART treatment. Regarding disclosure status, more than half, 349 (86.6%), were disclosed the disease to someone of which 142 (40.69%) defaulted from ART treatment. Considering tobacco and alcohol addiction patients, around 22.4%, and 68.5%, were tobacco and alcohol addicted patients respectively defaulted from ART treatment (Table 1).

**Table 1:** Baseline socio-demographic and behavioral characteristics of patients

Variables	Categories	Survival status		Total (%)
		Censored (%)	Event (%)	
Gender	Male	109 (76.22)	34 (23.78)	143 (35.5)
	Female	116 (44.62)	144 (55.38)	260 (64.5)
Age	15–24	55 (87.3)	8 (12.7)	63 (15.6)
	25–34	47 (25.82)	135 (74.18)	182 (45.2)
	35–44	72 (80.90)	17 (19.1)	89 (22.1)
	> 44	51 (73.91)	18 (26.09)	69 (17.1)
Residence	Urban	155 (70.45)	65 (29.55)	220 (54.6)
	Rural	70 (38.25)	113 (61.75)	183 (45.4)
Level of education	No Educated	36 (66.67)	18 (33.33)	54 (13.4)
	Primary	63 (45.32)	76 (54.68)	139 (34.5)
	Secondary	72 (55.38)	58 (44.62)	130 (32.3)
	Tertiary	54 (67.50)	26 (32.50)	80 (19.8)
Disclosure status	No	23 (33.33)	36 (66.67)	54 (13.4)
	Yes	207 (59.31)	142 (40.69)	349 (86.6)
Tobacco addiction	No	180 (52.17)	165 (47.83)	345 (85.6)
	Yes	45 (77.59)	13 (22.41)	58 (14.4)
Alcohol addiction	No	196 (63.02)	115 (36.98)	311 (77.2)
	Yes	29 (31.52)	63 (68.48)	92 (22.8)

Continuous variables	Minimum	Maximum	Mean	Standard error
Weight in kg	33.0	85.00	46.94	10.9
BMI in kg/m^2^	11.13	34.05	18.49	4.00

Out of 403 study participant incorporated in this study, those with a working functional status were 275 (68.2%) of which 87 (31.6%) defaulted from ART treatment. A large percentage of participants, 300 (74.4%), had good adherence status of which 41.7% defaulted from ART treatment during the follow-up period. Regarding the WHO clinical stage of participants, 224 (55.6%), 77 (19.1%), 74(18.4%), and 28 (6.9%) were stage-I, Stage-II, Stage-III, and Stage-IV, respectively. Similarly, the proportion of defaulter among HIV-TB co-infected participants was 83.9%. A significant proportion, of the study participants, 82 (20.3%), had one or more illnesses (opportunistic infections) other than TB, of which 55 (67.1%) had the outcome of defaulter. On the other hand, among the 89 (22.1%) participants who had affected by other comorbid condition (OCC) (Table 2).

**Table 2:** Base line clinical characteristics of patients

Variables	Categories	Survival status		Total (%)
		Censored (%)	Event (%)	
Functional status	Working	188 (68.36)	87 (31.64)	275 (68.2)
	Ambulatory	23 (22.77)	78 (77.2)	101 (25.1)
	Bedridden	14 (51.85)	13 (48.15)	27 (6.7)
Adherence	Poor	7 (43.75)	9 (56.25)	16 (4.0)
	Fair	43 (49.43)	44 (50.57)	87 (21.6)
	Good	175 (58.33)	125 (41.67)	300 (74.4)
WHO clinical stage	Stage-I	114 (50.89)	110 (49.11)	224 (55.6)
	Stage-II	53 (68.83)	24 (31.17)	77 (19.1)
	Stage-III	47 (63.51)	27 (36.49)	74 (18.4)
	Stage-IV	11 (39.29)	17 (60.71)	28 (6.9)
TB screen	No	216 (62.25)	131 (37.75)	347 (86.1)
	Yes	9 (16.07)	47 (83.93)	56 (13.9)
OIs	No	198 (61.68)	123 (38.32)	321 (79.7)
	Yes	27 (32.93)	55 (67.07)	82 (20.3)
OCC	No	203 (64.65)	111 (35.35)	314 (77.9)
	Yes	22 (24.72)	67 (75.28)	89 (22.1)

Continuous variables	Minimum	Maximum	Mean	Standard error
Hematocrit in %	20.80	54.30	37.66	4.50
White blood cell in 10^3^/$\mu l$	1.60	13.00	6.01	1.88
Red Blood Cell in 10^6^/$\mu l$	2.00	11.00	4.17	1.35
Platelet in 10^3^/$\mu l$	23	641	286.93	95.25
Lymphocyte in %	15.20	69.10	42.69	11.32
Monocyte in %	2.0	13.4	7.33	3.13

### Survival status of participants

Of 403 adult HIV patients followed for 72 months, 178 (44.2%) defaulted from ART treatment, while the remaining 255 (55.8%) were censored. The overall mean and median survival time was 44.3 and 42 months respectively (Table 3).

**Table 3:** Survival Status of patients

Survival status		Mean		Median	
Censored	Event	Estimate	Standard Error	Estimate	Standard Error
225 (55.8%)	178 (44.2%)	44.266	1.421	42.000	3.366

### Kaplan–Meier curve for some covariates

**Fig. 1:**
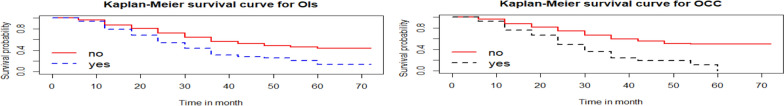
Kaplan–Meier Survival curve for OIs and OCC

Kaplan–Meier curves for some of the covariates like OIs and OCC of patients are shown in Fig. 1. The survival time of patients’ with-out OIs and OCC was higher than those with OIs and OCC, which suggested that these groups are at a lower risk of default from treatment than their counterparts (Fig. 1).

### Cox proportional hazards model

There is strong evidence that covariates residence, and TB screen had violated the assumption of PHs. However, the global test with 29 degrees of freedom was statistically insignificant at 5% level of significance. Therefore, there is a strong evidence of non-proportional hazards for residence, and TB screen. To accommodate the non-proportional hazard we divide the data into strata based on residence, and TB screen. Finally, each of the covariates, and the GLOBAL test with 27 degrees of freedom was not statistically significant. Therefore, the result showed that the Cox PHs assumption was satisfied. As a result, we have used the Cox PHs model, to analyze survival time to default.

### Cox PH model fitting

The covariates gender, disclosure, alcohol addiction, tobacco addiction, BMI, weight, WHO, adherence, OIs, OCC, hematocrit, platelet, and lymphocyte are statistically significantly in the univariable Cox PH model analysis associated with time to default from ART treatment, whereas age in years, educational status, functional status, monocyte, residence, TB, WBC, and RBC insignificant at a 25% level of significance. All the selected univariable covariates reanalyze using a survival sub model.

### Exploring smooth profile plot and the mean structure for CD4 cell count and hemoglobin

**Fig. 2:**
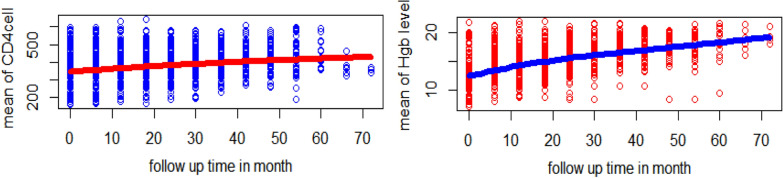
Smooth profile plot and the evolution of mean structure for CD4 Cell and hemoglobin level

Data exploration was engaged for repeatedly measurements of CD4 cell count, and hemoglobin levels before directly fitting the LMEM. Smooth profiles plot with the mean structure for CD4 cell, and hemoglobin level was used to isolate the general trend over time and provide information about the change at given times. Figure 2 indicates the two longitudinal responses increased through the visiting time.

### Model comparisons for linear mixed effect model

Different candidate model with different random effect were considered for CD4 count and hemoglobin level by using deviance information criteria (DIC). Hence, model-IV (random intercept and slope model) for both repeated measure responses in the full model was well-fitted (Table 4).

**Table 4:** Random effect model selection for repeated measure CD4 count and hemoglobin level

Model	Response variable	Random effect model		Null model	Full model
		Intercept only model	Intercept and slope model	DIC	DIC
I.	CD4 cell	CD4 cell	–	37,579.69	37,624.62
	Hgb level	Hgb level	–		
II.	CD4 cell	Hgb level	–	37,341.81	37,850.55
	Hgb level	–	CD4 cell		
III.	CD4 cell	CD4 cell	–	37,800.62	37,931.88
	Hgb level	–	Hgb level		
IV.	CD4 cell	–	CD4 cell	**37,329.52**	**37,092.11**
	Hgb level	–	Hgb level		

Bold values indicate better smallest DIC values

*CD4* cluster differentiation 4, *Hgb* *hemoglobin*, *DIC* deviance information criteria

### Linear mixed effect model fitting

At 25% level of significance the covariates gender, weight, functional status, adherence, WHO clinical stage, OIs, OCC, hematocrit, platelet, and lymphocyte are statistically significant in the univariable linear mixed effect model analysis associated with CD4 cell count. Likewise, gender, weight, BMI, functional status, adherence, WHO clinical stage, OIs, OCC, hematocrit, platelet, and lymphocyte are statistically, associated with Hemoglobin level. However, the remaining unselected covariates were insignificant at 25% level of significance.

### Bayesian joint longitudinal-survival sub-model analyses

Joint models explicitly link CD4 cell count and Hemoglobin level with time to-default through association parameters. Since, the posterior estimates of the association parameters in the joint analysis are statistically different from zero, providing strong evidence of association between the longitudinal and survival sub models. Hence, the two sub-models were related by applying communal covariates. The joint model is indicated in Tables 5 and 6.

**Table 5:** Bayesian joint model parameter estimates for longitudinal sub-model

Variable	Categories	CD4 cell count			Hemoglobin level		
		Posterior mean	Standard error	P-value	Posterior Mean	Standard error	P-value
Intercept	–	19.3132	0.3011	0.040	12.9976	0.1100	0.000
Visit time	–	1.4098	0.0047	0.000	0.1219	0.0002	0.000
Hematocrit	–	4.4815	0.0269	0.000	0.0084	0.0017	0.001
Weight	–	0.8874	0.0395	0.001	0.0183	0.0013	0.001
BMI	–	–	–	–	0.3242	0.0330	0.022
Platelet	–	0.1059	0.0012	0.016	0.0030	0.0000	0.002
Lymphocyte	–	0.7128	0.0110	0.040	0.0086	0.0030	0.001
Gender (Ref = male)	Female	0.3255	0.0057	0.001	0.2361	0.0105	0.001
Adherence (Ref = Poor)	Fair	7.4442	0.2183	0.270	0.4478	0.0114	0.001
	Good	6.6919	0.2198	0.001	0.4228	0.0085	0.018
Functional status( Ref = Working)	Ambulatory	− 2.5168	0.2157	0.730	− 0.0851	0.0078	0.001
	Bedridden	− 4.4877	0.2642	0.001	0.4902	0.0113	0.168
OIs (Ref = No)	Yes	− 1.0188	0.2232	0.601	− 0.2035	0.0071	0.001
OCC( Ref = No)	Yes	− 2.0880	0.2186	0.774	− 0.0620	0.0073	0.001
WHO stage (Ref = Stage-I)	Stage-2	0.0060	0.2093	0.000	0.1634	0.0078	0.492
	Stage-3	− 3.4512	0.2130	0.586	0.0532	0.0079	0.026
	Stage-4	− 3.3557	0.2801	0.001	− 0.8183	0.0116	0.018

**Table 6:** Bayesian joint model parameter estimates for survival sub-model

Variables	Categories	Posterior mean	Standard error	HR	95% CI for HR		p-value
					Lower	Upper	
Hematocrit	–	0.0157	0.0015	1.0158	1.0128	1.0188	0.001
BMI	–	0.0498	0.0081	1.0511	0.0191	1.1294	0.221
Platelet	–	0.0532	0.0054	1.0546	1.0435	1.0658	0.001
Weight	–	0.0800	0.0040	1.0833	1.0748	1.0918	0.000
Lymphocyte	–	0.0641	0.0042	1.0602	1.0575	1.0750	0.041
Sex (Ref = male)	Female	0.4085	0.0044	1.5046	1.1474	1.9975	0.004
Adherence (Ref = Poor)	Fair	0.0734	0.0041	1.0762	0.4874	3.4384	0.508
	Good	1.2329	0.0047	3.4312	2.4006	4.9392	0.000
WHO (Ref = Stage-I)	Stage-II	− 0.4223	0.0053	0.6555	0.3421	0.8891	0.002
	Stage-III	− 0.5700	0.0047	0.5655	0.0949	0.2734	0.000
	Stage-IV	− 0.2498	0.0064	0.7789	0.7692	0.7888	0.000
OIs (Ref = No)	Yes	0.1442	0.0039	1.1551	1.0908	1.4854	0.000
OCC(Ref = No)	Yes	0.0880	0.0038	1.0919	0.0839	1.1002	0.108
Disclosure (Ref = No)	Yes	− 0.3333	0.0045	0.7166	0.5458	0.9514	0.010
Tobacco (Ref = No)	Yes	− 0.5034	0.0047	0.6045	0.5989	0.6101	0.012
Alcohol (Ref = No)	Yes	− 0.3281	0.0036	0.7203	0.7152	0.7254	0.020
${\alpha }_{1}$	–	0.0037	0.0001	1.0037	1.0035	1.0039	0.001
${b}_{1}$	–	0.0021	0.0010	1.0021	1.0001	1.0041	0.004
${\alpha }_{2}$	–	0.4634	0.0014	1.5895	1.3975	1.8175	0.000
${b}_{2}$	–	0.0013	0.0001	1.0013	1.0011	1.0015	0.001

${\alpha }_{1}$ and ${\alpha }_{2}$ represents the association parameter of random intercept for CD4 and Hemoglobin, ${b}_{1}$ and ${b}_{2}$ indicates the association parameter of random slope for CD4 and Hemoglobin, CI credible interval and ref is reference category

Tables 5 and 6 indicate that female HIV positive patients had 0.326 per mm^3^ and 0.236 g/dl increment in their average CD4 cell count and Hemoglobin level than male patients respectively, by keeping all other covariates constant. Similarly, female patients had more hazard of defaulting (HR = 1.5) than male patients. Good adherence patients had increased the average CD4 cell count by 6.7 per mm^3^ and fair adherence patients had increased the average Hemoglobin level by 0.45 g/dl as compared to poor adherence patients. Similarly, good adherence Patients had more hazard of defaulting (HR = 3.43) than poor adherence patients. The remaining joint predictors can be similarly interpreted as above. Hence, as hematocrit, weight, platelet cell count, and lymphocyte count of a patient increased, CD4 cell count, Hemoglobin level, as well as its defaulted time increased. However, advanced clinical stage of patients was low CD4 cell count, Hemoglobin level and time to default.

## Discussions

In our study Hematocrit was found to be a significant predictor of CD4 cell count, Hemoglobin level and time to default from ART treatment in HIV positive individuals. Hematocrit increased by one unit, the average CD4 cell count had increased by 4.5 per mm^3^. Similarly, Hematocrit could be attributable to the maximizing Hemoglobin level and defaulters of patients.

Weight had a significant effect on CD4 cell count, Hemoglobin level and time to default from treatment. As weight of patients increased by one unit, the average CD4 cell count had increased by 0.9 per mm^3^.This result had been oppose by former study [23]. A unit increment of weight, the average blood Hemoglobin level increased by 0.02 g/dl. Weight of patients increases, the hazard of defaulter also increased by 8.3%. Our findings is consistence with a study conducted by [11]. However, this findings is contradict with another study conducted in Bahir Dar, Ethiopia [32]. This might be due to differences in the study area, sample size, and study period.

Results of the current study revealed that platelet cell count and lymphocyte count had an increment effects on CD4 cell count, and Hemoglobin level. Similarly, both predictors also increased by one unit, the hazard of defaulter were increased. This means adult HIV positive patients who had higher platelet cell count and lymphocyte count would have a higher CD4 cell count and Hemoglobin level with different increment of defaulter rates.

In this study female patient is a statistically significant factor for CD4 cell count. The result of this findings support the previous literature [11, 33]. The result of this study also shows that female patients increase the risk of defaulting as compared to male patients. the finding of this result is contradict with previous literature [32]. In their finding, the defaulting rate of female patients from the ART treatment had decreased as compared to male patients. This might be due to differences in the study area and period, sample size, and statistical model analysis.

The patient’s treatment adherence was also found to be a significant factor for CD4 cell count, Hemoglobin level and time to default. The patient’s repeated measure and survival time appears to increase with their level of treatment adherence. This could be patients may have better get of their health status related with increment of CD4 cell count, Hemoglobin level and decides to default from treatment easily.

In terms of WHO clinical stage; clinical stage-IV (severe disease) patients is lower CD4 cell count than clinical stage-I patients. This study supported by previous study in their result shown WHO clinical stage IV patients had a lower CD4 cell count than stage I during the follow-up period [34]. Clinical stage-II (mild disease) patients is a higher CD4 cell count than clinical stage-I patients. This result is also supported with a study done at Ethiopia [33]. WHO clinical stage-IV patient is also lower Hemoglobin level than clinical stage-I patient. Similarly, WHO clinical stage of adult HIV positive patients is a significant risk factor with time to default. This study was consistent with another study conducted by [32].

In joint model the estimates of the association parameter due to the slope of CD4 cell count is positive $$\left({b}_{1}=0.0021\right)$$, and Hemoglobin level ($${b}_{2}=0.0013)$$. similarly the association parameter for values of CD4 cell count ($${\alpha }_{1}=0.0037$$) and Hemoglobin level is($${\alpha }_{2}=0.463)$$ positive. This positive value represents direct relationship between the CD4 cell count, Hemoglobin level, and time to default.

This study indicating patients CD4 cell count and Hemoglobin level concentration increases from visit to visit, due to this increment variation patents default from treatment in different reason like patients might be feel better health condition (better quality of life) and might decide to default from treatment.

## Conclusion and recommendation

This study revealed that high hematocrit, high weight, high platelet cell count, high lymphocyte count, female patients, and good treatment adherence had joint significant predictors on adult HIV patients’ by increment of CD4 cell count, Hemoglobin level, and time to default from ART treatment. While, advanced WHO clinical stage-IV had joint significant predictors on adult HIV patients’ by decrement of CD4 cell count, Hemoglobin level, and time to default from ART treatment. To improve their health and extend their lives, patients with HIV should be given special attention based on these important factors. This study result can be used as bench mark for policymakers and concerned health staff to be considered for application of health related issues. It is recommended that further studies should be done on HIV positive adult patients by considering other important covariates that were not included in this study.

### Limitation of the study

This study was based on retrospective cohort study design, the data obtained from adult HIV positive patients chart. However, some important socio-demographic and clinical predictors like nutritional status, income status, homeownership, viral load count and other hematological parameters like eosinophil, neutrophil, and basophil counts, were not available on patient’s chart at study period (Additional file 1).

### Supplementary Information

**Additional file 1: Table 1.** Proportional assumption check.

## Data Availability

The data used in the current investigation is available from the corresponding author and can be attached upon request. The data accessed in the current investigation complied with relevant data protection and privacy regulations.

## Abbreviations

BMI: Body Mass Index
CD4: Cluster Differentiation 4
DIC: Deviance Information Criterion
E.C: Ethiopian Calendar
Hgb: Hemoglobin
HIV: Human Immune Deficiency Virus
LMEM: Linear Mixed Effect Model
OCC: Other Comorbid Condition
OIs: Opportunistic Infections
PHs: Proportional Hazards
PLWHIV: People Living With Human Immune Deficiency Virus
RBC: Red Blood Cell
UGSCH: University of Gondar Comprehensive Specialized Hospital
UNAIDS: The Joint United Nations Program on HIV/AIDS
WBC: White Blood Cell
WHO: World Health Organization
