# Supplementary material for: Predictors for CD4 cell count and hemoglobin level with survival time to default for HIV positive adults under ART treatment at University of Gondar Comprehensive and Specialized Hospital, Ethiopia
Source: BMC Res Notes. 2023 Dec 2;16:357. doi: 10.1186/s13104-023-06625-3 (PMC10693704; doi:10.1186/s13104-023-06625-3)
Supplement: Supplementary file 1 — Additional file 1: Table 1. Proportional assumption check. [file 13104_2023_6625_MOESM1_ESM.docx]

Table below explained the covariates that indicated proportional assumption with its Chi-Square statistics, Degree of freedom, p-value, and GLOBAL test

| Variables | Chi-Square statistics | DF | p-value | Variables | Chi-Square statistics | DF | p-value |
| --- | --- | --- | --- | --- | --- | --- | --- |
| Hematocrit | 0.2281 | 1 | 0.633 | Adherence | 5.0036 | 2 | 0.082 |
| Weight | 0.6352 | 1 | 0.425 | WHO stage | 1.7258 | 3 | 0.631 |
| BMI | 2.6652 | 1 | 0.103 | Functional status | 0.3804 | 2 | 0.827 |
| WBC | 2.5015 | 1 | 0.114 | OIs | 0.3492 | 1 | 0.555 |
| RBC | 0.2212 | 1 | 0.638 | OCC | 0.1645 | 1 | 0.685 |
| Platelet | 0.0102 | 1 | 0.920 | Education | 2.4215 | 3 | 0.490 |
| Lymphocyte | 0.2584 | 1 | 0.611 | Disclosure | 4.7404 | 1 | 0.209 |
| Monocyte | 0.4648 | 1 | 0.495 | Tobacco addiction | 3.8517 | 1 | 0.5100 |
| Sex | 1.0264 | 1 | 0.311 | Alcohol addiction | 0.5709 | 1 | 0.450 |
| Age | 4.2007 | 3 | 0.241 | **GLOBAL** | **36.6008** | **27** | **0.103** |

Key: DF; Degree of freedom
